# NADomics: Measuring NAD^+^ and Related Metabolites Using Liquid Chromatography Mass Spectrometry

**DOI:** 10.3390/life11060512

**Published:** 2021-05-31

**Authors:** Nady Braidy, Maria D. Villalva, Ross Grant

**Affiliations:** 1 Centre for Healthy Brain Ageing, School of Psychiatry, University of New South Wales, Sydney, NSW 2052, Australia; m.villalva@unsw.edu.au; 2 Euroa Centre, UNSW School of Psychiatry, NPI, Prince of Wales Hospital, Barker Street, Randwick, Sydney, NSW 2031, Australia; 3 School of Medical Sciences, University of New South Wales, Sydney, NSW 2052, Australia; r.grant@unsw.edu.au; 4 Australasian Research Institute, Sydney Adventist Hospital, Sydney, NSW 2076, Australia

**Keywords:** NAD^+^, nicotinamide, ageing, plasma, biomarker

## Abstract

Nicotinamide adenine dinucleotide (NAD^+^) and its metabolome (NADome) play important roles in preserving cellular homeostasis. Altered levels of the NADome may represent a likely indicator of poor metabolic function. Accurate measurement of the NADome is crucial for biochemical research and developing interventions for ageing and neurodegenerative diseases. In this mini review, traditional methods used to quantify various metabolites in the NADome are discussed. Owing to the auto-oxidation properties of most pyridine nucleotides and their differential chemical stability in various biological matrices, accurate assessment of the concentrations of the NADome is an analytical challenge. Recent liquid chromatography mass spectrometry (LC-MS) techniques which overcome some of these technical challenges for quantitative assessment of the NADome in the blood, CSF, and urine are described. Specialised HPLC-UV, NMR, capillary zone electrophoresis, or colorimetric enzymatic assays are inexpensive and readily available in most laboratories but lack the required specificity and sensitivity for quantification of human biological samples. LC-MS represents an alternative means of quantifying the concentrations of the NADome in clinically relevant biological specimens after careful consideration of analyte extraction procedures, selection of internal standards, analyte stability, and LC assays. LC-MS represents a rapid, robust, simple, and reliable assay for the measurement of the NADome between control and test samples, and for identifying biological correlations between the NADome and various biochemical processes and testing the efficacy of strategies aimed at raising NAD^+^ levels during physiological ageing and disease states.

## 1. Introducing NADomics as a Tool for Quantification of the NADome in Biological Samples

**Figure 1:**
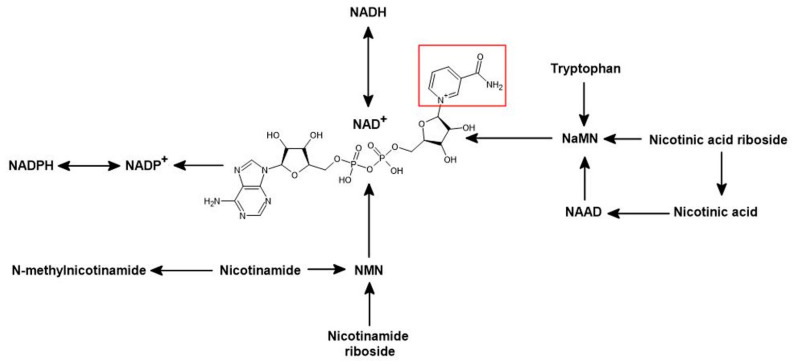
NAD^+^ metabolism in eukaryotic cells. NAD^+^ anabolism from tryptophan occurs by the de novo kynurenine pathway (KP). NAD^+^ precursors via the salvage pathway include nicotinamide (NAM), nicotinic acid (NA), nicotinamide riboside (NR), and nicotinic acid riboside (NAR). The enzyme nicotinamide phosphoribosyltransferase (NAMPT) converts NAM to nicotinamide mononucleotide (NMN). Nicotinamide mononucleotide adenylyltransferase (NMNAT1-3) converts NMN to NAD^+^. NAM can be methylated to N-methyl-nicotinamide (MeNAM) by the action of nicotinamide N-methyltransferase (NNMT). NADH represents the reduced form of NAD^+^. NADP^+^ is the phosphorylated form of NAD^+^. NADP^+^ can be reduced to NADPH by NAD kinases (NADK1,2). PARPs, Sirtuins, and CD38 NAD^+^ glycohydrolases are known as NAD^+^ consumers, leading to the generation of NAM. Nicotinic acid phosphoribosyltransferase (NAPRT) converts NA to nicotinic acid mononucleotide (NAMN), which is then converted to NAD^+^ by NMNAT1-3. NAR needs to be converted to NAMN to yield NAD^+^ synthesis via nicotinamide riboside kinases (NRK1,2). NRK1,2 also convert NR to NMN. NAR can form NA via purine nucleoside phosphorylase (PNP). PNP are also capable of converting NR to NAM.

NADomics is the high-throughput study of nicotinamide adenine dinucleotide (NAD^+^) and its related metabolites. NAD^+^ is an essential coenzyme that is present in all organisms [1]. NAD^+^ serves as a major coenzyme for enzymatic reduction–oxidation reactions and ATP production. More recently, NAD^+^ has also been shown to be a crucial co-substrate for numerous enzymes (i.e., sirtuins, NAD^+^ glycohydrolase (CD38), poly(adenosine diphosphate–ribose) polymerases (PARPs)) [2,3,4]. The term NADomics is an analogy to metabolomics, the study of the metabolome. The word NADome is a portmanteau of NAD^+^ and its related metabolome. The NADome is the entire set of NAD^+^ metabolites that are anabolised or catabolised by an organism or system (Figure 1). The emerging field of NADomics has enabled the identification and quantification of ever-increasing numbers of NAD-related metabolites.

NAD^+^ and its reduced form NADH can be phosphorylated to NADP^+^ and NADPH, which serve as major coenzymes in over 400 oxidoreductase enzymes [5]. NAD^+^ serves as a hydrogen acceptor allowing the transfer of electrons for oxidation–reduction (i.e., redox) reactions leading to ATP production in the mitochondria [1]. NAD^+^ glycohydrolases (CD38, CD157) are involved in the production of calcium-mobilising messengers, ADP-ribose (ADPR) and its cyclic form (cADPR) [6]. PARP-mediated ADP-ribosylation uses the ADPR moiety of NAD^+^ to repair DNA, leading to the breakdown NAD^+^ to nicotinamide (NAM) and an ADP-ribosyl product [7]. Sirtuins are a family of class III NAD^+^-dependent histone deacetylases that exhibit protein lysine deacetylase, and partial ADPR transferase activities [8]. Deacetylation occurs when the modified lysine side chain is coupled to the cleavage of the glycosidic bonds in NAD^+^, leading to the generation of the deacetylated lysine, acetylated ADPR, and NAM as by-products [9]. These processes are dependent on NAD^+^ availability, and NAM is an endogenous inhibitor of CD38, PARP, and sirtuins [5].

Continuous replenishment of cellular NAD^+^ levels is important for normal cellular survival [10]. The de novo NAD^+^ biosynthesis pathway in most cells is dependent on the amino acid tryptophan via the kynurenine pathway. When the availability of dietary tryptophan is limited, the efficiency of the conversion of tryptophan to NAD^+^ decreases below the well-established conversion ratio of 60:1 [11,12]. Nicotinic acid (NA), NAM, NAM riboside (NR), or NA riboside (NAR) can also be used to synthesise NAD^+^ via the NAD^+^ salvage pathway [5]. NAM can be methylated to N-methyl-nicotinamide (MeNAM) by the action of NAM N-methyltransferase [13]. The enzymes nicotinamide phosphoribosyltransferase (NAMPT) and nicotinic acid phosphoribosyltransferase (NAPRT) convert NAM and NA to nicotinamide mononucleotide (NMN) and nicotinic acid mononucleotide (NAMN), respectively [5]. Additionally, the phosphorylation of nicotinamide riboside (NR) and nicotinic acid riboside (NAR) via nicotinamide riboside kinase (NRK) also leads to the production of NMN and NAMN, which can be converted to nicotinic acid adenine dinucleotide (NAAD) and NAD^+^ by nicotinamide mononucleotide adenyltransferase (NMNAT) [14]. NAAD can be amidated to NAD^+^ by NAD synthetase (NADS) [5].

The NADome is important in physiological processes such as energy production, transcriptional regulation, DNA repair, protein modification, and secondary messenger signalling [5]. Therefore, the application of NADomics in the clinic provides an essential indicator of nutritional status, redox function, and incidence and progression of age-related diseases [15]. A decline in cellular NAD^+^ levels has been associated with mitochondrial impairments, immune dysfunction, and reduced histone deacetylase activity, which can interfere with several transcription factors and affect gene expression levels [5]. Reduced NAD^+^ levels also have a dramatic impact on the activity of PARPs, thus impairing DNA repairing. We and others have demonstrated that intracellular NAD^+^ levels decline in conditions of metabolic stress in muscle, brain, heart [2], lung [2], liver [2], kidney [2], skin [16], and plasma [17,18] in humans and rats. NAD^+^ levels are reduced in tissues and cells exposed to oxidative stress and DNA damage, the overfed liver, the failing heart, the damaged peripheral neuron, and the injured brain [5], and correlate with disease severity of multiple sclerosis [19]. However, it remains unclear whether a depressed NADome is a function of age, although ageing is a major risk factor for the accumulation of metabolic stress.

Promotion of cellular NAD^+^ anabolism has been shown to restore NAD^+^ levels and reverse some phenotypes of ageing by enhancing cellular repair and stress resistance. Recent studies have shown that administration of the NAD^+^ precursors, nicotinamide riboside (NR) and nicotinamide mononucleotide (NMN), can attenuate pathology in several murine models of age-related disorders [20]. Additionally, oral administration of NR has been reported to increase whole blood NAD^+^ levels in humans [21]. A 6-week randomised, double-blind, placebo-controlled crossover clinical trial reported that NR supplementation was well-tolerated and effectively promoted NAD^+^ metabolism in healthy middle-aged and elderly adults [22]. However, another randomised, placebo-controlled, double-blinded trial showed that NR supplementation had no effect on insulin sensitivity and lipid mobilising effects, and no adverse events due to NR were reported [23]. We also demonstrated that intravenous (I.V) injection of NAD^+^ increased plasma and urine NAD^+^ levels and exhibited differential effects on the NADome in healthy middle-aged humans. Thus, while it can be argued that there are several strategies to increase NAD^+^ levels, understanding the endogenous intracellular and extracellular levels of the NADome are of emerging interest.

## 2. Clinical Relevance for Detection of the NADome in Biological Fluids

The NADome is an essential mediator of metabolic pathways that have been associated with ageing and age-related disorders. Several published methods have identified the NADome in whole blood and various cells or tissues, however, very few studies have examined changes in the NADome extracellularly [17,18,24]. A previous study reported that NAD^+^ is predominantly an intracellular nucleotide, and only phosphorylated metabolites could be detected using LC-MS/MS [21]. However, emerging evidence suggests that exogenous NAD^+^ may cross the plasma membrane and replenish intracellular NAD^+^ levels in mammalian cells [25,26]. The discrepancy is likely to be related to the differences in the LC-MS/MS methodology and sample extraction. For example, Trammell et al. [27] used a double extraction method and two different chromatographic runs for reduced and oxidised forms of adenine and pyridine nucleotides, extending the analysis time per sample.

**Figure 2:**
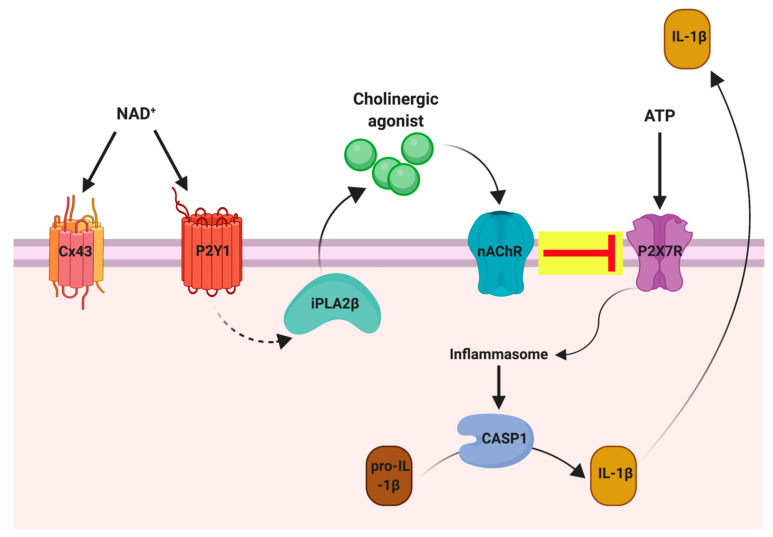
Schematic representation of the role of NAD^+^ in purinergic signalling. Extracellular NAD^+^ and ATP are released from damaged cells. ATP binds to the ATP-sensitive P2X7 receptor of monocytic cells. Activation of inflammasomes and caspase-1 induces cleavage of pro-IL-1β and release of bioactive IL-1β. NAD^+^ binds to P2Y receptors and activates iPLA2β, leading to the production and release of bioactive mediators which serve as nicotinic agonists.

Owing to the clinical significance of increasing NAD^+^ levels, and the role of NAD^+^ in several age-related degenerative diseases, analytical methods for quantification of the NADome can be applied to clinically relevant biological samples, including whole human blood, urine, and CSF. This is because: (1) NAD^+^ is released from cells at low amounts, (2) NAD^+^ catabolism is an immediate process that leads to production of biologically active products, including NAM and consequently, MeNAM, (3) NAD^+^ can act directly on cell surface receptors such as connexin 43 channels and purinergic P2 receptors and therefore must be present extracellularly (Figure 2) [28], (4) MeNAM is the main metabolite of pyridine nucleotide catabolism and is cleared by urinary excretion [29], and (5) there is a lack of NAD-consuming enzymes in the urine compared to plasma.

NAD^+^ and related metabolites present in the blood will be filtered at the glomerulous and will be present in at least the renal filtrate of the proximal tubule. Recent studies have shown that CD38-mediated cADPR production in renal arteries and the distal tubule is necessary for Ca2+-mediated regulation of renal function [30]. Thus, the presence of CD38, as a NAD-dependent ectoenzyme in the distal renal tubule, and which its activity is critical for normal renal function and dependent on the availability of NAD^+^ as a substrate, strongly supports the view that intact NAD^+^ is still present in the renal filtrate after most of the tubular reabsorption has occurred.

A unique element of the central nervous system (CNS) is its high oxygen consumption and energy requirements relative to size (i.e., 2% of body weight and uses 20% of oxygen). This high energy demand is vital to sustaining the complex metabolic activities of the CNS and is achieved through accelerated mitochondrial activity using readily available NAD^+^ in its redox couple. Importantly, elevated mitochondrial activity also results in significant superoxide production and nuclear damage [31], necessitating DNA repair through PARP activity, a NAD^+^ catabolising process. In addition to its role in intracellular metabolism, extracellular NAD^+^ also exerts direct synaptic effects, reducing synaptic excitotoxicity [32], and may also act as a neurotransmitter [33]. In order to act at the synapse, extracellular NAD^+^ is clearly present in the CNS, as recently reported [33].

NAD^+^ is also a central player in the maintenance and control of biological rhythms coordinated through the 20,000 pacemaker neurons in the suprachiasmatic nucleus in the CNS, where NAMPT/NAD+ drives the circadian clock feedback cycle through SIRT1 and CLOCK:BMAL1 [34]. Numerous NADPH-diaphorase (i.e., nitric oxide-producing) neurites are present on the free surface of the ependymal layer in direct contact with the cerebrospinal fluid (CSF) [35], thus suggesting that NADPH must also be present in the CSF to engage with this enzyme. Additionally, nicotinamide N-methyltransferase is present in the CSF and actively converts CSF NAM into its N-methylated metabolite [36], further highlighting both the presence and potential importance of NAM for the CNS.

## 3. Analysis of the NADome Using Traditional Techniques

Several methodologies have been previously used to quantify the NADome. These include specialised HPLC-UV, NMR [37], capillary zone electrophoresis, or colorimetric enzymatic assays [38]. While commonly available biochemical assays are readily available and relatively inexpensive, they provide indirect measurements and require tedious enzymatic manipulation [39,40], limiting their use in the clinic. Moreover, these assays are only available for NAD^+^, NADH, and ATP, and thus are unable to provide an accurate reflection of changes in other metabolites in the NADome, such as NADP^+^ and NADPH. For instance, the intracellular levels of NAD^+^ have been shown to vary between 1 µM to 1 mM [16]. In addition, the ratio of NADPH:NADP^+^ was reported to be ~100 using an enzymatic assay which measures the substrate concentration of malic and isocitrate dehydrogenase enzymes [41]. However, direct measurement of these metabolites suggests that the ratio of NADPH:NADP^+^ may be significantly lower, i.e., 3.3 and 0.04 in the rat liver and heart [42]. Although multiple values are missing in the current literature and biological variability may indeed play a role, it is more likely that analytical variation represents a major contributory factor to the reported differences.

NMR-based experimental approaches have been developed to quantify the NADome in human cells. ^1^H NMR spectroscopy has been previously used to quantify the NADome in human platelets and erythrocytes [43]. While the relative concentration can be quantified directly from the NMR spectrum for each metabolite, irrespective of the complexity of the sample, this approach may require up to 2 h of data acquisition to achieve a signal-to-noise ratio of at least 5 for a sample containing approximately 2 µM of metabolite [43]. This can limit the application of NMR for the quantification of the NADome in some biological specimens, where some metabolites may be present in very low nanomolar levels [15].

Liquid chromatography (LC) techniques using absorbance for detection provide some advantages for quantifying multiple metabolites in the NADome relatively fast, i.e., within 10 to 60 min. However, HPLC-UV methods are limited due to low sensitivity and the presence of co-eluting contaminants [16]. For example, in a complex biological sample, a single peak may represent the metabolite of interest and other related metabolites that share identical retention times. Some studies have used mass spectrometry to non-quantitatively confirm the nature of the metabolite(s) present in any given fraction [16]. However, this strategy is time-consuming and costly. Since many metabolites in the NADome can be converted into other metabolites either by oxidation/reduction or enzymatic reactions, accurate quantification of NAD^+^, NMN, NAM, and other metabolites without examination of the entire NADome may be misleading.

## 4. Quantification of the NADome Using Liquid Chromatography-Mass Spectrometry

**Figure 3:**
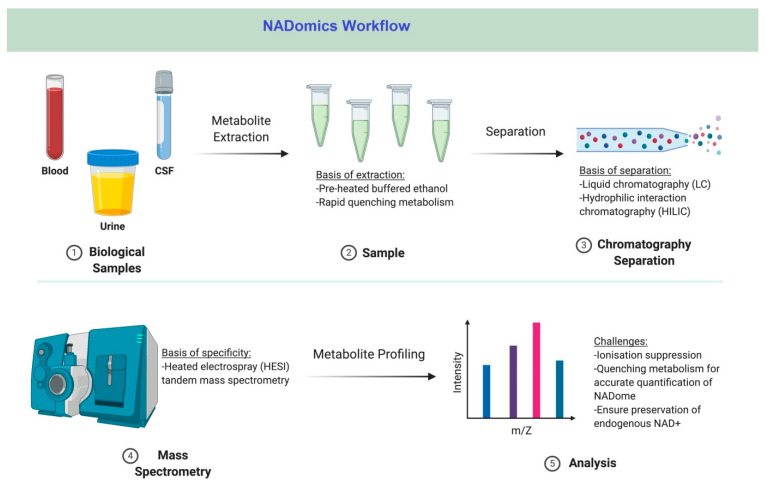
NADomics workflow. The NADomics workflow involves profiling the NADome with statistically significant variations in biological samples, e.g., blood, urine, and CSF. The specific NAD^+^ metabolite ID including chemical structure and concentrations can be elucidated using LC-MS/MS. Analysis is the final step to elucidate associations between the identified metabolite and its role in physiology and disease.

LC coupled to tandem mass spectrometry (LC-MS/MS) has been recently developed for the quantitation of the NADome in biological specimens (Figure 3) [16]. In line with HPLC assays, LC allows the separation of individual metabolites and must be optimised in a similar manner to HPLC methods. The data collected from LC-MS is two-dimensional, i.e., retention time and mass-charge ratio, thus increasing specificity and sensitivity and allowing for the separation of closely related metabolites such as NAD^+^ and NADH. Additionally, most LC-MS assays have a limit of quantification in the femtomole range [16].

Hydrophilic interaction liquid chromatography (HILIC) is an emerging separation mode of LC that suits well for the quantification of the NADome. The variant uses polar columns with a stationary phase, whereby polar analytes are eluted from the column by increasing water content of the mobile phase (typically acetonitrile with low amounts of water). HILIC also allows for hydrogen donor interactions between neutral polar species, and weak electrostatic mechanisms under high organic solvent conditions [44]. The retention of the analytes, peak shape, and chromatographic tailing is regulated by the pH of the mobile phase and ion strength attributed to ionic additives such as ammonium acetate and ammonium formate. HILIC allows for a high flow rate due to very low column backpressure by the high organic mobile phase [44].

HILIC separation has been previously used for the quantification of AMP, GMP, UMP, CMP, and IMP in infant formula [45,46]. It has also been adapted for quantitative analysis of cAMP, ATP, and other nucleosides, and mono-, di-, and tri-phosphate nucleotides, thus allowing for the simultaneous analysis of a large number of metabolites in a single run [45,46]. A volatile additive in mobile phase enables smooth hyphenation with mass spectrometry detection and has recently been optimised for the detection of at least 17 different metabolites in the NADome in astrocytes and oocytes [47].

## 5. Challenges of NADomics in Biological Specimens

Accurate quantification of the NADome is crucial for evaluating the cellular redox status. In this section, we discuss potential challenges and solutions that have affected previous methods.

### 5.1. Extraction

Extraction of the NADome is a major source of analytical variation. Immediate extraction of the NADome in biological samples is ideal. Extraction methods which fail to inactivate enzyme activities following cell lysis can limit the accurate quantitation of the NADome. For example, the levels of NAD^+^ in a biological sample can be degraded to 1% of the anticipated value, while the levels of NAM can increase more than 10-fold. The most common method of extraction for most NAD^+^ metabolites is boiled buffered ethanol [16]. We previously demonstrated that ice-cold 80% methanol was suitable for the extraction and maintaining molecular integrity of the NADome in murine oocytes [47].

A recent study compared the quenching and extraction efficiency of 7 different solvents on the NADome in mammalian cells and mouse tissue [42]. The solvents included a cold aqueous buffer with/without detergent, hot aqueous buffer, cold organic mixtures such as 80% methanol, buffered 75% acetonitrile, and acidic 40:40:20 acetonitrile:methanol:water with 0.02 or 0.1 M formic acid. The study found that extraction with acidic 40:40:20 acetonitrile:methanol:water was most efficient at maintaining the NADome for measurement using LC-MS. However, inclusion of detergent may also be useful, albeit to a lesser extent [42].

Human plasma, serum, urine, and cerebrospinal fluid (CSF) require only the removal of proteins, which can be performed using pre-heated buffered ethanol solution (ethanol:HEPES 1 mM, pH 7.1) [24], ice-cold methanol [17], methanol:acetonitrile, or centrifugal filtration apparatus. Additionally, a recent method quantified the NADome in human cell cultures, erythrocytes, CSF, and primate skeletal muscle without drying steps (using steam drying or speed vac), thus increasing NADome stability [24].

### 5.2. Internal Standards

Ionisation suppression is a major problem that should be considered when measuring the NADome. This phenomenon refers to the ability of some sample components to influence the ionisation and detectability of certain analytes [16]. Therefore, the peak height or peak area may not be a true reflection of the peak size in the original complex mixture. Hence, internal standards are necessary to minimise ionisation suppression errors [16]. Optimisation of internal standards is also important to minimise inaccuracies in the quantification of the NADome due to interconversion of some metabolites, i.e., non-enzymatic degradation limits the accurate measurement of the NAD^+^:NADH and NADP^+^:NADPH ratios. Spiking with internal standards can monitor interconversion of these metabolites [42].

Previous studies that quantified the NADome using LC-MS/MS assays used internal standards derived from yeast cultured in ^13^C-glucose-supplemented (^13^C ^1^^5^N)-labelled medium [48] or in ^13^C-glucose with excellent correlation results [16]. However, yeast cell culture facilities may not always be available in-house. Isotopic labels for some NAD^+^ metabolite isotopic labels are not commercially available. In the absence of the exact isotypic label, a closely related molecule (structural analog) is recommended [49]. Evans [50] quantified 18 metabolites of the NADome with excellent correlation coefficients using external standards.

We previously used the following internal standards for the quantification of selected NAD^+^ metabolites in human and murine cells: ^2^H₄-NAM (MeNAM, NAM, NA, NAMN, NADPH, NAAD, ADPR, cADPR), ^13^C₅-Adenosine (adenosine), ^13^C₅-Cyclic AMP (cAMP, NAD^+^, NMN), ^13^C₁₀^1^^5^N₅-ATP (NADH, ATP), ^13^C₅-AMP (AMP), and ^1^^5^N₅-ADP (NADP^+^, ADP). Most of these internal standards displayed correlation coefficients (r^2^ ≥ 0.98) [47].

### 5.3. Analyte Stability

Instability of pyridine nucleotides is likely to be the biggest challenge when quantifying the NADome in a variety of biological samples. For instance, while reduced forms (i.e., NADH and NADPH) are more stable in alkaline solution, the oxidised forms (NAD^+^ and NADP^+^) are more stable in acidic solutions, and this is likely due to acid-catalysed autoxidation of NADH and NADPH [27]. Since time, pH, and temperature are likely to have a major effect on the ability to accurately quantify the NADome, quick quenching of metabolism is essential.

Demarest et al. recently assessed the benchtop stability of the NADome in human red blood cells (RBCs), the epithelial cell line HEK-293T, and primate skeletal muscle [24]. The study demonstrated rapid degradation of NADH and NADH within 30 min in the cellular matrices, whereas NAD^+^ and ADPR were only stable for 10 min, and only 20% remained after 30 min. On the other hand, NAM and NMN levels increased after 1 h. This may be due to increased catabolism and degradation of NAD^+^ and other metabolites [24]. Interestingly, NMN levels decreased in RBCs and skeletal muscle, while NADP^+^ remained stable for 1 h in both RBCs and HEK-293T cells. In the CSF, only NAD^+^ and NMN could be detected in the linear range. NAD^+^ demonstrated greater stability in the CSF compared to the cellular matrices and this was attributed to a reduced NADome in the CSF and/or limited availability of NAD^+^ ‘consumers’, e.g., PARPs and Sirtuins [24].

Therefore, optimising sample collection, storage, and availability of suitable testing protocols is essential to retain and accurately report changes to the NADome in intervention studies. In addition, quenching of samples should be completed without delay to minimise degradation of metabolites by active enzymes and preserve the endogenous NADome.

### 5.4. Liquid Chromatography

Perhaps the most well-described LC-MS assay for quantification of the NADome is based on hydrophilic interaction liquid chromatography. One method to quantify the NADome uses two different mobile phases on two porous graphitic carbon reversed phases (Hypercarb, Thermo) in alkaline (NMN, NAMN, ADP, ATP, NAD^+^, NADH, NAAD, NADP^+^) and acid separation (NA, NAM, and NR) [16]. We previously demonstrated that an amino column using a dual HILIC-RP gradient with heated electrospray (HESI) tandem mass spectrometry detection in mixed polarity multiple reactions monitoring (MRM) mode could be simultaneously used for the quantification of the NADome in a single chromatographic run in biological specimens [47]. Recently, another study using an Accucore HILIC column identified some metabolites in multiple transitions [24]. For instance, NAD^+^ was observed in the NAAD transition. Additionally, NAM was observed in the NA transition, but these metabolites could not be resolved. Picolinic acid, a metabolite in the de novo NAD^+^ synthesis pathway and an isomer of NA, also co-eluted with NA [24].

Our refined method is an application of hydrophilic interaction chromatography (HILIC)—a major chromatographic system used in metabolic profiling [50]. The retention mechanism is based on partitioning and water is used as the eluent. An amino-modified HILIC Phenomenex Luna NH_2_ column has been previously shown to demonstrate good retention and chromatographic resolution of water-soluble metabolites, including the NADome, with good peak shape, compared to cyano and/or silica columns, and none of the selected metabolites were observed in multiple MRM transitions [47,50].

## 6. Concluding Remarks

It is well-established that concentrations of the NADome represent a useful marker for elucidating the current status of cells, and may likely be an important biomarker in several metabolic and age-related disorders. Thus, accurate quantification of the NADome may be beneficial for researchers in understanding the pathobiology of metabolic disorders and effects of drug candidates. LC-MS/MS represents a rapid, robust, simple, and reliable assay for the measurement of the NADome in clinically relevant biological tissue. LC-MS/MS requires minimal sample processing. Using the amino phase chromatographic separation and commercially available internal standards eliminates cost, and requirement for yeast cultures for labelled metabolites, which are likely to represent major obstacles for measurement of the NADome to be applied in clinical diagnosis. NADomics can be used to provide renewed insights on physiological and pathological processes and may assist in identifying and evaluating potential therapeutic strategies.

## Data Availability

Not applicable.
